# Regorafenib inhibited gastric cancer cells growth and invasion via CXCR4 activated Wnt pathway

**DOI:** 10.1371/journal.pone.0177335

**Published:** 2017-05-10

**Authors:** Xiao-Lin Lin, Qi Xu, Lei Tang, Li Sun, Ting Han, Li-Wei Wang, Xiu-Ying Xiao

**Affiliations:** Department of Oncology, Ren Ji Hospital, School of Medicine, Shanghai Jiao Tong University, Shanghai, People's Republic of China; National Cancer Center, JAPAN

## Abstract

**Aim:**

Regorafenib is an oral small-molecule multi kinase inhibitor. Recently, several clinical trials have revealed that regorafenib has an anti-tumor activity in gastric cancer. However, only part of patients benefit from regorafenib, and the mechanisms of regorafenib’s anti-tumor effect need further demonstrating. In this study, we would assess the potential anti-tumor effects and the underlying mechanisms of regorafenib in gastric cancer cells, and explore novel biomarkers for patients selecting of regorafenib.

**Methods:**

The anti-tumor effects of regorafenib on gastric cancer cells were analyzed via cell proliferation and invasion. The underlying mechanisms were demonstrated using molecular biology techniques.

**Results:**

We found that regorafenib inhibited cell proliferation and invasion at the concentration of 20μmol/L and in a dose dependent manner. The anti-tumor effects of regorafenib related to the decreased expression of CXCR4, and elevated expression and activation of CXCR4 could reverse the inhibition effect of regorafenib on gastric cancer cells. Further studies revealed that regorafenib reduced the transcriptional activity of Wnt/β-Catenin pathway and led to decreased expression of Wnt pathway target genes, while overexpression and activation of CXCR4 could attenuate the inhibition effect of regorafenib on Wnt/β-Catenin pathway.

**Conclusions:**

Our findings demonstrated that regorafenib effectively inhibited cell proliferation and invasion of gastric cancer cells via decreasing the expression of CXCR4 and further reducing the transcriptional activity of Wnt/β-Catenin pathway.

## Introduction

Of all the cancers, gastric cancer ranks the fourth and the fifth respectively among males and females worldwide in terms of incidence rate, while it ranks the third and the fifth respectively in terms of mortality rate [1]. Most patients are either diagnosed at an advanced stage, or develop a relapse after surgery with curative intent. Moreover, gastric cancer has a high rate of recurrence and metastasis, and most patients have a low 5-year survival rate [2–4]. So there is a clear and emergency need for new treatment regimens. Despite recent advances in adjuvant/neo-adjuvant therapy and improved understanding of gastric cancer biology, progress in the treatment of gastric cancer has been limited. Compelling data have emerged to improve the prognosis of advanced gastric cancer, and increased attention has been given to the use of small-molecule inhibitor in gastric cancer therapy, recently.

Regorafenib is an orally administered small-molecule inhibitor of multiple protein kinases. Preclinical data showed that regorafenib inhibited tumor angiogenesis, stroma formation and also tumor cells growth through targeting VEGFRs (vascular endothelial growth factor receptors) 1, 2 and 3, tyrosine-protein kinase receptor TIE-2, PDGFR (platelet-derived growth factor receptor)-β, FGF (fibroblast growth factor) receptor 1, proto-oncogene tyrosine-protein kinase receptor Ret, mast/stem cell growth factor receptor Kit and RAS/RAF/MEK/ERK pathway, proto-oncogene serine/threonine-protein kinase B-raf [5–8]. However, the mechanisms of regorafenib inhibiting cancer cells have not fully understood. Clinical studies have demonstrated that regorafenib exhibited broad antitumor activity in a series of solid tumors. Phase Ⅲ studies have showed that regorafenib significantly improved overall survival (OS) and Progression-Free-Survival (PFS) in patients with metastatic colorectal cancer and advanced gastrointestinal stromal tumors (GIST) [9–10]. Although a number of clinical studies have shown that anti-angiogenesis inhibitors such as bevacizumab and sunitinib in the treatment of gastric cancer have limited efficacy [11–14], recently, a phase Ⅱ trial (INTEGRATE) showed that regorafenib prolonged PFS in refractory advanced gastric adenocarcinoma and the phase Ⅲ trial is planned [15]. However, the trial also showed that regorafenib was only effective in about 40% of patients with gastric cancer. The fact reveals that the resistance to regorafenib readily appears(PFS:2.6 months)and 32% of patients had at least one serious adverse event in the regorafenib group, so the overall clinical efficacy of regorafenib remains quite limited [15–16]. But the mechanism of resistance to regorafenib has not been clearly understood. Therefore, investigation of the mechanism of resistance and biological marker predicting the efficiency of regorafenib for gastric cancer would be significantly valuable for the application of regorafenib.

In our study, we found that the anti-tumor effects of regorafenib correlated with CXCR4 levels in gastric cancer cells, and CXCR4 further reduced the transcriptional activity of Wnt/β-Catenin pathway. Our findings revealed that CXCR4 might mediate the anti-tumor effect of gastric cancer to regorafenib, and might be a novel biomarker for patients selecting of regorafenib.

## Materials and methods

### Cell culture and reagents

The human gastric cancer cell lines MKN-28, SGC7901, and MKN-45 were purchased from American Type Culture Collection (ATCC) and cultured in RPMI 1640 supplemented with 10% fetal bovine serum (FBS), 1% glutamax, and 1% P/S and maintained in an incubator with a humidified atmosphere of 5% CO_2_ at 37°C. The RPMI1640 and FBS were purchased from Life Technologies.

### Cell proliferation assay

The cells were seeded in a 96-well plate at a concentration of 5×10^3^ cells/well a day before the experiment. 3-[4,5-Dimethylthiazol-2-yl]-2,5- diphenyltetrazolium bromide (MTT, 0.5 mg/ml, Sigma, St. Louis, MO, USA) was added to each well 1, 2, 3, 4, and 5 days after seeding. Cells were cultured at 37°C for 4 h, followed by addition of 150 ml DMSO. Absorption was measured at a wave length of 490nm.

### Soft agar assay

The cells were seeded in six-well plates for the soft agar assay. Each well contained a bottom layer of 1.2% agarose, a middle layer of 0.6% agarose that included 3000 cells, and a top layer of medium, which was changed every sixth day. After 25 days, the colonies were counted by Quantityone analysis software (BioRad Inc., Hercules, CA, USA).

### Invasion assay

The transwell invasion assay was performed using a Millicell invasion chamber (Millipore, Billerica, MA, USA). The 8-μm pore inserts were coated with 15 μg of Matrigel (Becton Dickinson Labware, Bedford, MA, USA), and 5×10^4^ cells were seeded in the top chamber. The Matrigel invasion chamber was incubated for 24 h in a humidified tissue culture incubator. Non-invading cells were removed from the top of the Matrigel with a cotton-tipped swab. Invading cells on the bottom surface of the filter were fixed in methanol and stained with crystal violet. Invasion ability was determined by counting the stained cells.

### Reverse transcription and quantitative real-time PCR

Total RNA was isolated by Trizol (Takara Biotechnology Co. Ltd., Dalian, China). cDNA synthesis was conducted as followed with the SYBRs ExScriptt RT-PCR kit (Takara Biotechnology Co. Ltd., Dalian, China) according to manufacturer’s instruction. The quantitative real-time PCR were employed using the ABI PRISM 7300 Sequence Detection System (Applied Biosystems, Foster City, CA, USA). Relative quantification of gene expression was determined using the comparative C_T_ method. Gene expression levels in A cells relative to B cells were calculated using the following formulas: ΔΔCT = ΔCT A-ΔCT B, fold change = 2^-ΔΔCT^.

### Protein extraction and western blotting

Total protein was isolated from 5×10^6^ cells with 200 ml of ice-cold lysis buffer containing 1% Nonidet P-40 (NP-40), 50 mmol/l Tris (pH 7.4), 150 mmol/l NaCl, 0.1% sodium dodecyl sulfate (SDS), 0.5% deoxycholate, 200 mg/ml phenylmethanesulfonyl fluoride (PMSF), and 50 mg/ml aprotinin. Insoluble materials were removed by centrifugation at 20000 g for 20 min at 4°C. Clarified protein lysates (50 μg) were electrophoretically resolved on a denaturing SDS polyacrylamide gel, and electrotransferred onto nitrocellulose membranes. The membranes were initially blocked with 5% nonfat dry milk in Tris-buffered saline (TBS) for 2 h and then probed with primary antibodies against CXCR4 (ab2074, abcam) and GAPDH (ab9485, abcam) as loading control. Immunodetection was carried out using the ECL Western Blotting Detection Kit (Amersham Corp, UK). Relative protein expression levels were quantified by densitometric measurement of ECL reaction bands and normalized to GAPDH levels.

### β-Catenin/Tcf transcription reporter assay

1×10^5^ cells were seeded per well in a 24-well plate in gastric cancer cell lines MKN-28, SGC7901, and MKN-45 before transient transfection with the construct TOPflash or FOPflash reporter plasmid (Millipore, Billerica, MA, USA). All transfections were performed using 0.8 mg of TOPflash or FOPflash plasmid and 2 ml lipofectamine 2000. To normalize the transfection efficiency in reporter assays, the cells were co-transfected with 0.02 mg of an internal control reporter plasmid, containing Renilla reniformis luciferase driven by the TK promoter. At 24 h after TOPflash or FOPflash transfection, the luciferase assay was performed with the Dual Luciferase Assay System kit (Promega Corp., Madison, WI, USA). Relative luciferase activity was reported as the fold induction after normalization for transfection efficiency.

### Lentivirus-mediated gene overexpression

Lentivirus-mediated overexpression of human CXCR4 (Genbank accession, no.NM_003467) was constructed using the pGLV5-EF1a-GFP vectors (GenePharma, Shanghai, China). Cells transductions were conducted by mixing virus with cells. Eight hours after transduction, the medium was changed, and cells were replenished with fresh medium. After a recovery period of 24h, puromycin (10μg/ml) was added to select cells with stable virus integration into the genome. Primers used are shown in Table 1.

**Table 1:** Primers of real-time PCR used in this study.

Name	Sequence (5’-3’)	Product size
CXCR4	F: CTGAGAAGCATGACGGACAAG	175
	R: GGATGAGGACACTGCTGTAGA	
CTNNB1	F: CGACACCAAGAAGCAGAGATG	193
	R: GAACTAGTCGTGGAATGGCAC	
CD44	F: CTTTCTGCACTATTCCCAGCC	241
	R: CTCTGGGAAAACAAGAGGCAC	
CD31	F: GCTGACCCTTCTGCTCTGTT	150
	R: TGAGAGGTGGTGCTGACATC	
CCND1	F: CCTGTCCCACTCCTACGATAC	171
	R: CCAAGTAGCTGTGGGTTGAAC	
GAPDH	F: CAAAAGGGTCATCATCTCTGCC	179
	R: TCATGAGTCCTTCCACGATACC	

### Statistical analysis

All statistical analyses were performed using the SPSS 19.0 software. The results were presented as means ± standard deviation (SD) of three replicate assays. Differences between the groups were assessed by the Student’s t-test. *P* < 0.05 was considered to indicate statistical significance.

## Results

### Regorafenib inhibited the growth of gastric cancer cells

**Fig 1:**
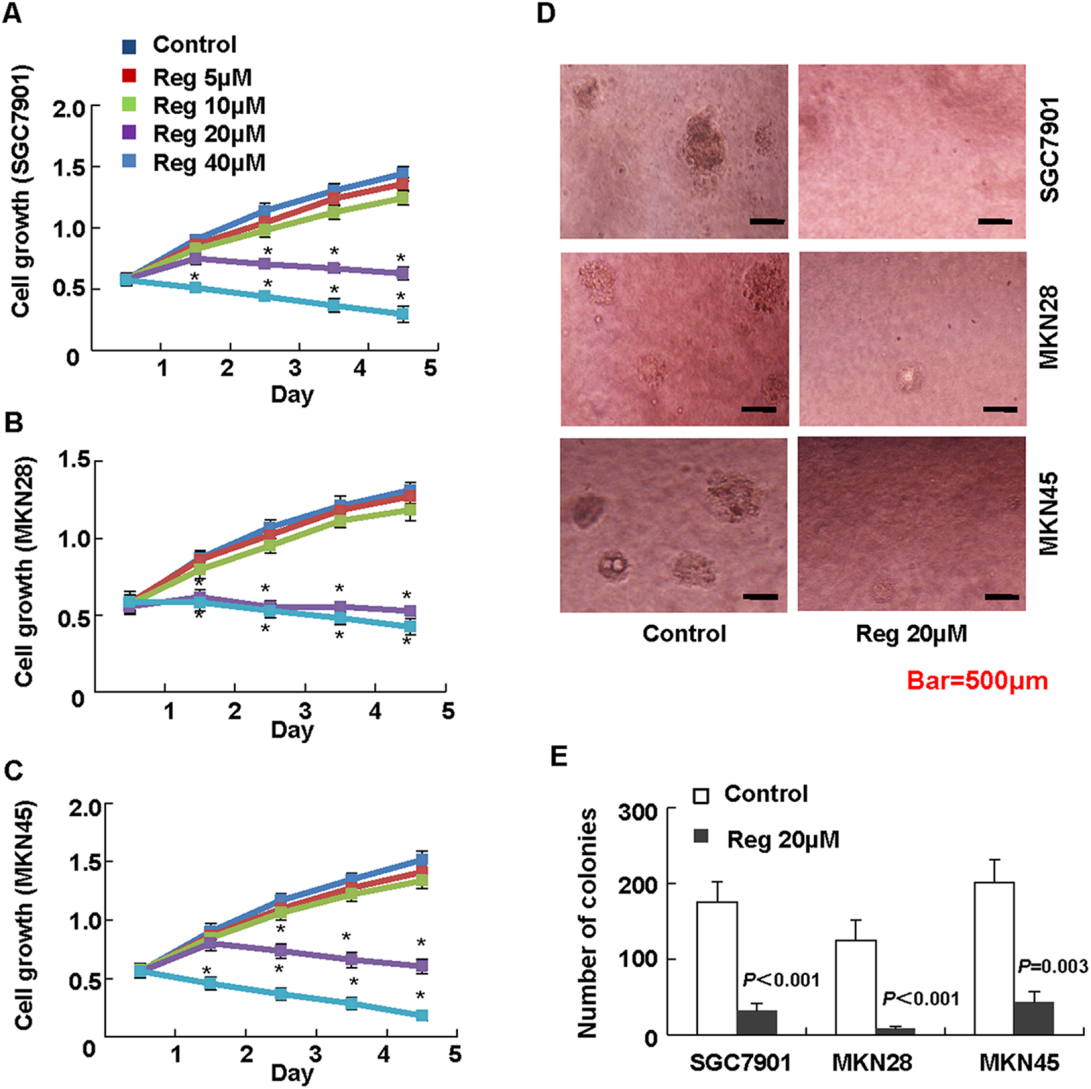
The inhibitory effect of regorafenib on the growth of gastric cancer cells. Cell lines SGC7901(A), MKN-28(B) and MKN-45(C) were seeded in 96-well plates. Cytotoxicity of regorafenib was assessed using MTT test at day1, day 2, day 3, day 4 and day 5 of different concentration groups. All assays were performed in triplicate. D and E, the cell colonies were significantly reduced in regorafenib groups (20μmol/L) in soft agar assay (*P*<0.05).

Firstly, we investigated the role of regorafenib on the anchorage-dependent growth of gastric cancer cells. MTT assay showed that treated with regorafenib at the concentration of 20μmol/L significantly inhibited gastric cancer cell growth as compared to control, and the inhibition effect showed dose dependent (Fig 1A–1C, S1 Table). Soft agar assay showed that regorafenib dramatically decreased the formation of colonies of SGC7901, MKN 28 and MKN45 (Fig 1D–1E, S2 Table). These results revealed that regorafenib inhibited the growth of gastric cancer cells.

### Regorafenib blocked the invasion of gastric cancer cells

**Fig 2:**
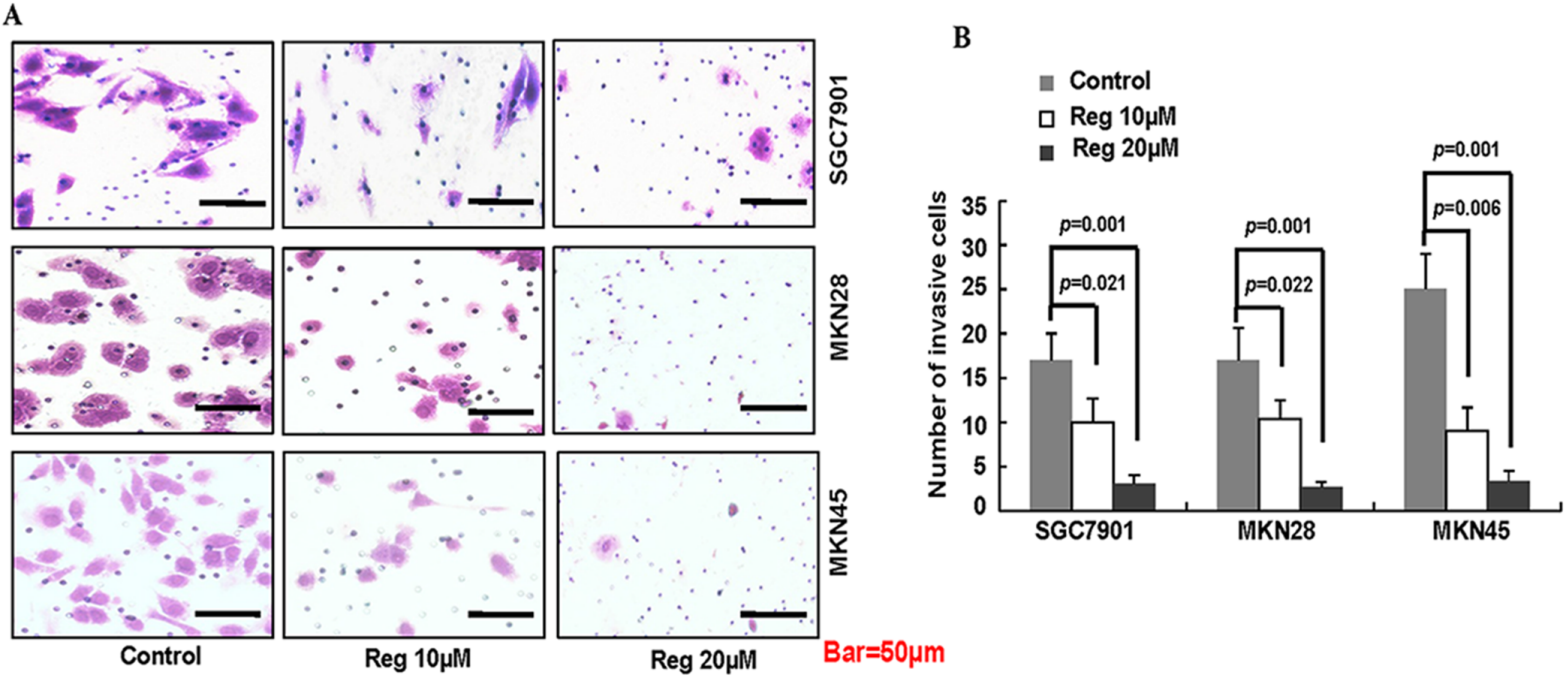
The inhibitory effect of regorafenib on the invasion of gastric cancer cells. The invasion of SGC-7901, MKN-28, and MKN-45 cells were determined as described in Materials and Methods. Representative tumor cell invaded were photographed (A) in a comparison of the control groups (B, *, *P*<0.05).

We then determined the effect of regorafenib on the invasion ability of gastric cancer cells via the Matrigel invasion assay. The results showed that treated with regorafenib at the concentration of 10μmol/L or 20μmol/L significantly decreased the invasion ability of SGC7901, MKN 28 and MKN45 as compared to the control groups (Fig 2A and 2B, S3 Table). The results revealed that regorafenib inhibited the invasion ability of gastric cancer cells.

### Regorafenib suppresses the expression of CXCR4 in gastric cancer cells

Chemokines and chemokine receptor 4 (CXCR4) plays an important role in gastric cancer growth, invasion and metastasis [17–19]. To determine how regorafenib affected proliferation and invasion in gastric cancer cells, we then investigated whether regorafenib modulated the expression of CXCR4 in gastric cancer.

**Fig 3:**
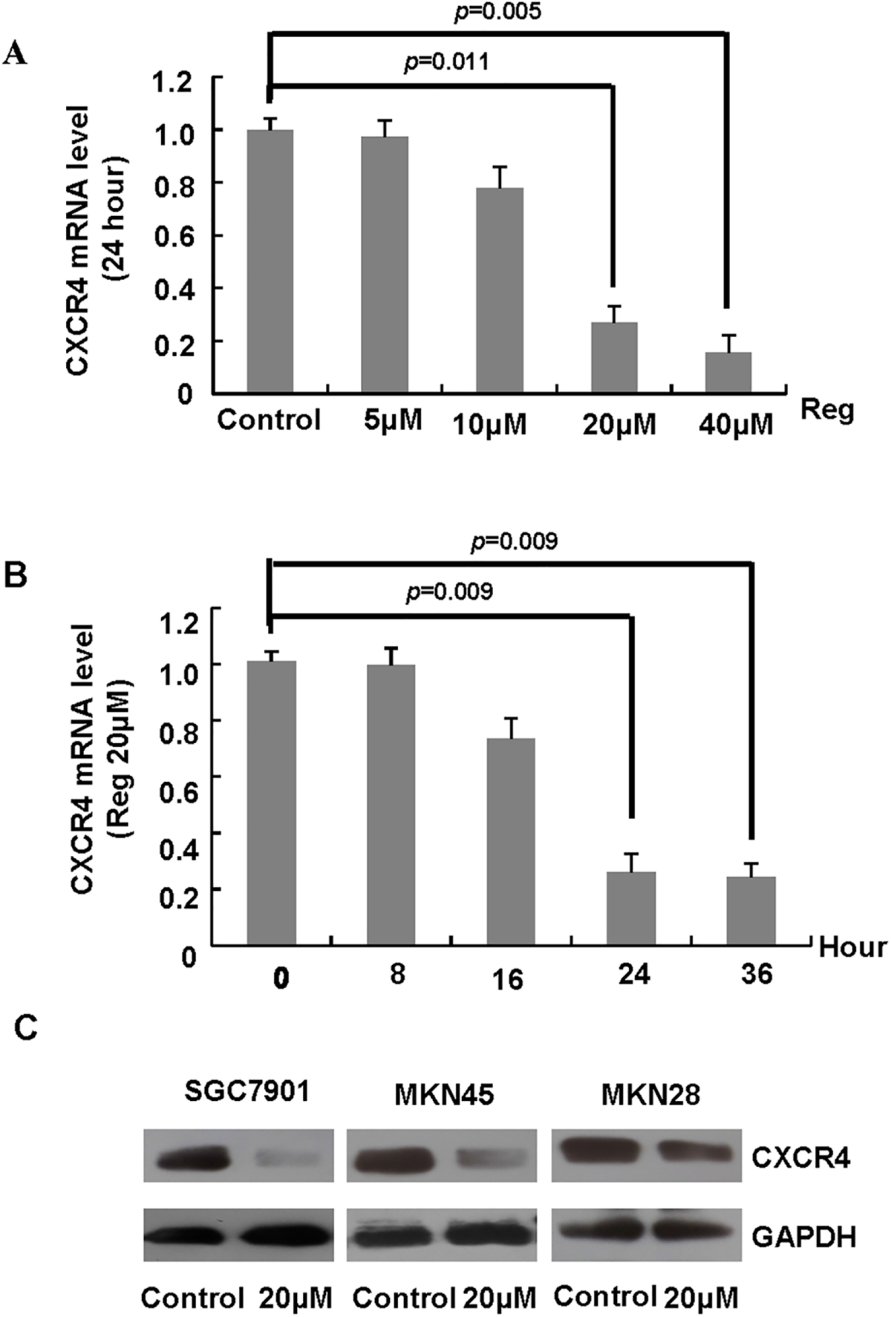
The effects of regorafenib on CXCR4 expression in gastric cancer cells. A and B, SGC7901 Cells were treated with regorafenib at different concentration (5μmol/L,10μmol/L,20μmol/L,40μmol/L) for 24 hours or at different times (0 hours, 8 hours,16 hours, 24 and 36 hours) at the concentration of 20μmol/L. The mRNA levels of CXCR4 were measured using real-time PCR. C. SGC-7901, MKN-28, and MKN-45 cells were treated with regorafenib at the concentration of 20μmol/L, and the protein levels of CXCR4 were analyzed via western blot.

The results of real-time PCR showed that regorafenib decreased the mRNA levels of CXCR4 in SGC7901 cells at 24 and 36 hours, and in a dose–dependent manner (Fig 3A and 3B, S4 Table). Furthermore, as shown in Fig 3C (S1 File), western blot analysis confirmed that treated with regorafenib at the concentration of 20μmol/L decrease the protein level of CXCR4 in gastric cancer cells. These data above demonstrated that regorafenib may inhibit the growth and invasion of gastric cancer cells via decreased the expression of CXCR4.

### Elevated expression and activation of CXCR4 reversed the inhibition effect of regorafenib on gastric cancer cells

**Fig 4:**
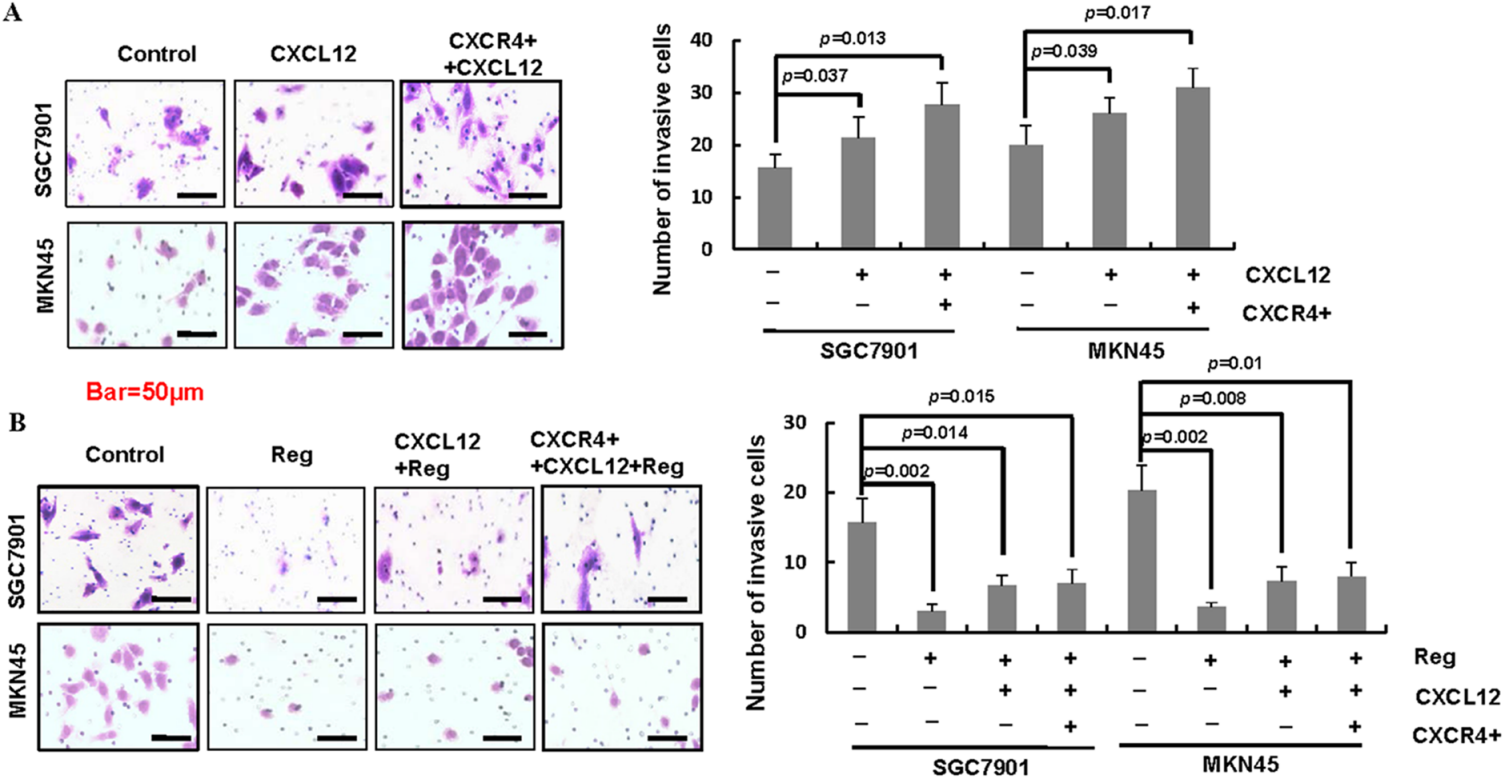
CXCR4 reversed the inhibition effect of regorafenib on gastric cancer cells. A. Gastric cancer cells treated with CXCL12 alone or treated with CXCL12 together with CXCR4 overexpression enhanced the invasion of gastric cancer cells compared to the control, and CXCL12 together with CXCR4 overexpression further increased the invasion of gastric cancer cells. B. Overexpression and activation of CXCR4 could reverse the inhibition effect of regorafenib in gastric cancer cells. (CXCR4+, CXCR4 overexpression).

The ligand of CXCR4 is CXCL12 (stromal cell-derived factor 1, SDF-1). The binding of CXCL12 to CXCR4 has been reported to play important roles in cancer growth, invasion and metastasis [20–21]. As showed in Fig 4A (S5 Table), CXCL12 enhanced the ability of invasion, and treated with CXCL12 and CXCR4 overexpression further increased the invasion of gastric cancer cells.

We have showed that regorafenib inhibited gastric cancer cells growth and invasion via downregulating the expression of CXCR4. Here, we further investigated whether overexpression and activation of CXCR4 could reverse the inhibition effect of regorafenib. As showed in Fig 4B (S5 Table), CXCR12 significantly abrogated the inhibitory effect of regorafenib, and CXCL12 together with CXCR4 overexpression further attenuated the inhibition of regorafenib in gastric cancer cell invasion. These results revealed that elevated expression of CXCR4 and activation of CXCR4 with its ligand CXCL12 abrogated the inhibition effect of regorafenib on gastric cancer cells.

### Regorafenib inhibited Wnt/β-catenin pathway via CXCR4

**Fig 5:**
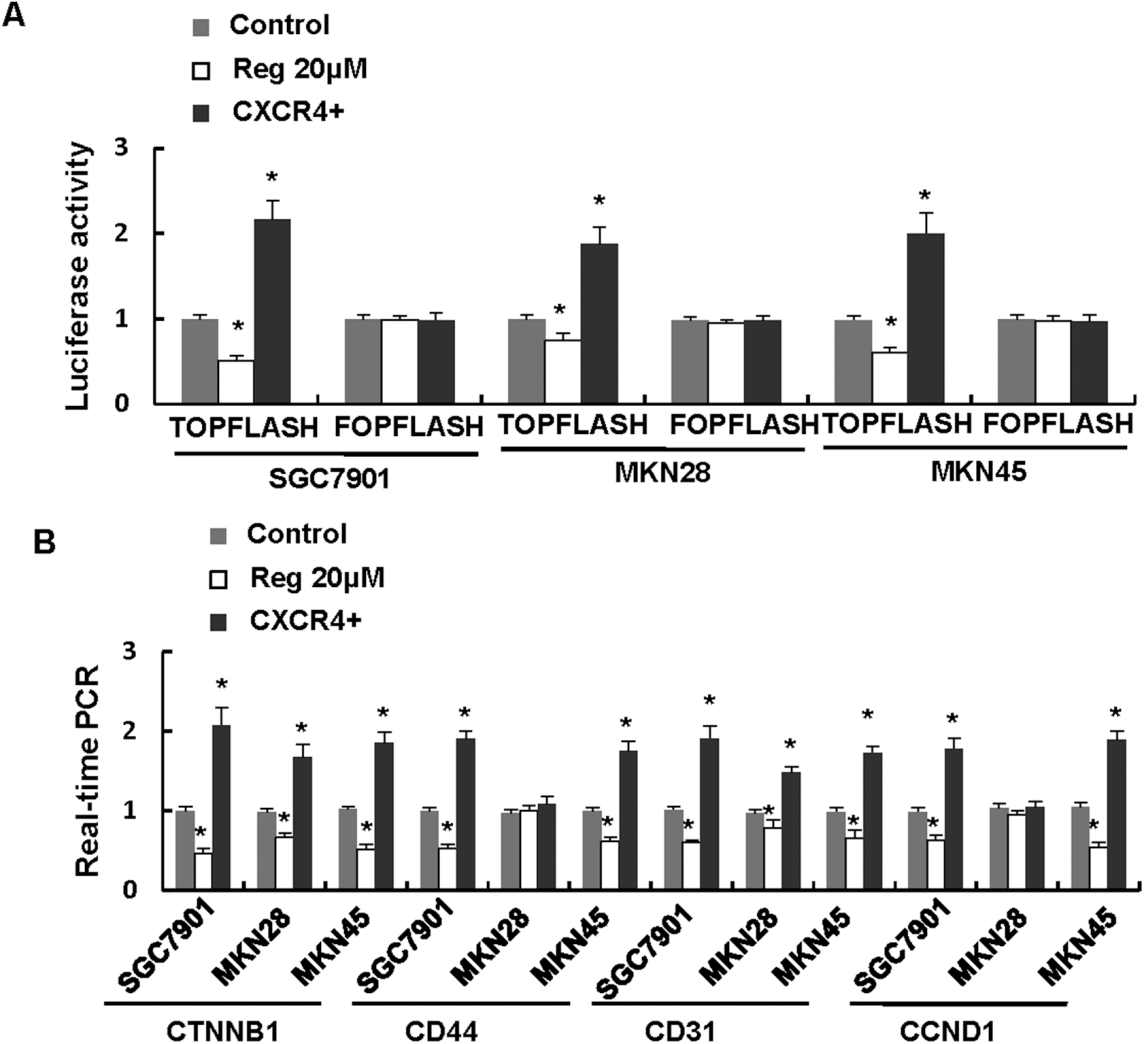
Regorafenib inhibited Wnt/β-catenin pathway via CXCR4. A. The activity of TOP and FOP flash were analyzed in gastric cancer cells treated with regorafenib and CXCR4 overexpression. B. Real-time PCR showed that the expression of Wnt target genes such as *CTNNB1*, *CD44*, *CD31* and *CCND1* were increase in gastric cancer cells with CXCR4 overexpression, whereas decreased in gastric cancer cells treated with regorafenib at the concentration of 20μmol/L as compared to the control.

It has been reported that Wnt/β-catenin pathway played critical roles in the development and progression in gastric cancer, and CXCR4 has been published as an upstream regulator of Wnt/β-catenin pathway. We then determined whether regorafenib inhibited and CXCR4 enhanced the Wnt/β-catenin signaling pathway in gastric cancer. The β-catenin/Tcf transcription reporter assay was recognised as an important assessment method for evaluation of the cardinal Wnt pathway activity. As TOP flash has three TCF-binding sites, it could be applied to represent the activation of the Wnt pathway. The results showed that compared to the control group, regorafenib significantly decreased the TOP flash activity. Nevertheless, overexpression of CXCR4 increased the TOP flash activity (Fig 5A, S6 Table). Furthermore, real-time PCR analysis showed that the expression of Wnt target genes such as *CTNNB1 (β-Catenin*), *CD44*, *CD31* and *CCND1*(*CyclinD1*) were decreased in gastric cancer cells treated with regorafenib at the concentration of 20μmol/L, but overexpression of CXCR4 increased the mRNA levels of Wnt target genes (Fig 5B, S7 Table). These data suggested that Wnt signaling pathway might be important for the inhibitory effect of regorafenib on gastric cancer cells, and regorafenib might inhibit the Wnt/β-catenin pathway via CXCR4 (S1 Fig).

## Discussion

The key part of the development and metastasis of gastric cancer is tumor angiogenesis which is regulated by interconnected signaling pathways [4, 5]. Vascular endothelial growth factor receptor (VEGFR) 2 which plays crucial roles in physiological and pathological angiogenesis of normal and tumor vasculature is the core target of ramucirumab, apatinib and regorafenib. Ramucirumab is a fully human IgG1 monoclonal antibody; its clinical activity as a second-line therapy for advanced gastric cancer was demonstrated in the REGARD study and RAINBOW study [22–23]. Apatinib treatment significantly improved the OS and PFS with an acceptable safety profile in patients with advanced gastric cancer refractory to two or more lines of prior chemotherapy in a Phase Ⅲ trial [24]. The development of more effective agents and the identification of biomarkers that can be used for the diagnosis, prognosis, and individualized therapy for gastric cancer patients, have the potential to improve the efficacy and safety for gastric cancer treatments [25]. The similarity of drug targets and the high reproducibility of clinical benefit not only validate the important role of anti-angiogenesis therapy in second and third line treatment of advanced gastric cancer [26], but also provide a basis for selecting the core target of anti-angiogenesis research of gastric cancer in the future. Regorafenib is a small-molecule inhibitor of multiple protein kinases and inhibited tumor angiogenesis through targeting VEGFR1, 2 and 3, PDGFR-β, FGFR1, Kit, RAS/RAF/MEK/ERK pathway and so on. Recent studies showed that regorafenib also inhibited cancer cell growth. In this study, we showed that regorafenib inhibited anchorage-dependent growth and invasion of gastric cancer cells. But the mechanism of regorafenib’s anti-tumor effect was not fully demonstrated in gastric cancer.

A phaseⅡtrial (INTEGRATE) has demonstrated that regorafenib was effective in prolonging PFS in refractory advanced gastric adenocarcinoma; and the preliminary test results of another ongoing trial also show that unresectable or metastatic esophagogastric (EG) cancer patients may benefit from the treatment of FOLFOX plus regorafenib [15, 27–28] (S8 Table). However, the clinical efficiency of regorafenib was limited because of the resistance of gastric cancer to regorafenib and the high incidence of side effects. Thus a better understanding of the mechanisms, which mediated the inhibitory effect of regorafenib on gastric cancer cells, would help us to understand the mechanism of the resistance to regorafenib and further discover the biomarkers that might identify the sensitive patients who would benefit from regorafenib.

Now the role of chemokines in tumor metastasis has become more and more important, and chemokines and their receptors play critical roles in cancer growth, invasion and metastasis [29]. CXCR4 is the one of the best known chemokine receptors. Jiang YX et al. investigated that invasive gastric CSCs were CD26+ and CXCR4+ and were closely associated with increased metastatic ability [30]. CXCR4 has been reported to be overexpressed in more than 20 different tumors, including gastric cancer. Qiao J, et al. found that SRF promote gastric cancer metastasis by facilitating myofibroblast-cancer cell crosstalk in an SDF1-CXCR4 dependent manner [17]. Guo ZJ et al. demonstrated that RUNX2 promotes the invasion and metastasis of human gastric cancer by transcriptionally up-regulating the chemokine receptor CXCR4 [31]. Also, Inflammatory cytokines VEGF and the growth factor receptor HER2 has also been correlated with overexpression of the chemokine receptor SDF-1, also known as CXCL12 can mediate angiogenesis via its cognate receptor CXCR4 [32]. The protein has 7 transmembrane regions and is located on the cell surface. Mutations in this gene have been associated with WHIM (warts, hypogammaglobulinemia, infections, and myelokathexis) syndrome. Detailed study of the underlying molecular mechanisms reveal that cancer cell CXCR4 overexpression contributes to aggressive tumor behavior, tumor growth, invasion, angiogenesis, metastasis, relapse, and therapeutic resistance [33–35], and upregulated expression of CXCR4 was an independent prognostic predictor for patients with gastric cancer [36]. CXCR4 is unique in that it exclusively interacts with the endogenous ligand CXCL12 [37]. CXCL12/CXCR4 signaling axis regulates the process of tumor proliferation and metastasis [20–21]. Studies have shown that blocking the CXCR4 can reduce proliferation and metastasis, and induce apoptosis of cancer cell [38–39]. In addition, CXCR4 has been found to be associated with tumor drug resistance [40–41], and Gao DY et al. found the clinical potential of CXCR4-targeted NPs for delivering sorafenib and overcoming acquired drug resistance in liver cancer [42] which suggesting that CXCR4 may be associated with anti-angiogenic drug resistance.

Then we investigated the effect of regorafenib whether or not related with CXCR4 expression. The results indicated that regorafenib decreased the expression of CXCR4 in a dose–dependent and a time–dependent manner in gastric cancer cells. Our data suggested that the potential underlying mechanism of the inhibition effect of regorafenib in gastric cancer cells was correlated with CXCR4. The CXCR4/CXCL12 axis plays an important role in tumorigenesis, metastasis, and recurrence of tumors. However, whether CXCR4/CXCL12 affected regorafenib sensitivity in gastric cancer cell has not been fully investigated. Our study assessed the correlation between CXCR4/CXCL12 and regorafenib sensitivity in SGC-7901 and MKN-45 cell lines. The data showed that CXCL12 treatment or CXCL12 treatment combined with CXCR4 overexpression reduced the anti-tumor effect of SGC-7901 and MKN-45 gastric cells to regorafenib. Our results suggested that CXCR4/CXCL12 might serve as a potential drug sensitivity biomarker of regorafenib.

Wnt/β-catenin pathway had proved to be a crucial pathway for gastric carcinogenesis [43–45], and Wnt pathway has been reported to be activated by CXCR4/CXCL12 [46]. We then investigated whether regorafenib affected Wnt/β-catenin pathway in a CXCR4/CXCL12-dependent manner. The data showed that regorafenib inhibited the Wnt/β-catenin pathway transcriptional activity and target genes such as CTNNB1, CD44, CD31 and CCND1. And overexpression of CXCR4 elevated the activity of Wnt pathway.

## Conclusion

In conclusion, our study demonstrates that regorafenib obtain obvious anti-tumor activity by inhibiting cell proliferation and invasion in gastric cancer cells. And our results revealed that the inhibitory effect of regorafenib was mediated by CXCR4. Furthermore, regorafenib also inhibit the Wnt/β-catenin pathway target genes in a CXCR4 dependent-manner. Our findings demonstrated that regorafenib inhibited gastric cancer cells proliferation and invasion via decreasing the expression of CXCR4 and further reducing the transcriptional activity of Wnt/β-Catenin pathway. We revealed the potential anti-tumor effects and underlying mechanisms of regorafenib in gastric cancer cells, and CXCR4 might mediated the anti-tumor effect of gastric cancer cell to regorafenib, and might be a novel biomarker for patients selecting of regorafenib. These results suggest that the CXCL12/CXCR4 signaling axis might play an important role in gastric cells resistance to regorafenib which will help to study other anti-vascular drug resistance mechanism.

## Supporting information

S1 Fig Regorafenib inhibited gastric cancer cells growth and invasion via CXCR4 activated Wnt pathway. (TIF)

S1 File Original uncropped and unadjusted gels/blots. (RAR)

S1 Table Cytotoxicity assessment of regorafenib on the growth of gastric cancer cells. (DOC)

S2 Table Cell colonies in regorafenib groups (20μmol/L) in soft agar assay. (DOC)

S3 Table Number of invasive cells with regorafenib at the concentration of 10μmol/L or 20μmol/L. (DOC)

S4 Table The mRNA levels of CXCR4 of SGC7901 Cells treated with regorafenib at different concentration for 24 hours or at different times at the concentration of 20μmol/L. (DOC)

S5 Table Number of invasive cells with different test treatments. (DOC)

S6 Table The activity of TOP and FOP flash in gastric cancer cells treated with regorafenib or CXCR4 overexpression. (DOC)

S7 Table The expression of Wnt target genes in gastric cancer cells with CXCR4 overexpression or treated with regorafenib. (DOC)

S8 Table Clinical outcomes of recent trials of regorafenib in advanced gastric and gastroesophageal junction cancer. (DOC)

## References

[1] Torre LA, Bray F, Siegel RL, Ferlay J, Lortet-Tieulent J, Jemal A. Global cancer statistics, 2012. CA Cancer J Clin. 2015;65(2):87–108. doi: 10.3322/caac.21262 25651787 10.3322/caac.21262

[2] Shah MA, Ajani JA. Gastric cancer: an enigmatic and heterogeneous disease. JAMA.2010;303(17):1753–1754. doi: 10.1001/jama.2010.553 20442394 10.1001/jama.2010.553

[3] Cristescu R, Lee J, Nebozhyn M, Kim KM, Ting JC, Wong SS, et al Molecular analysis of gastric cancer identifies subtypes associated with distinct clinical outcomes. Nat Med. 2015;21(5):449–456. doi: 10.1038/nm.3850 25894828 10.1038/nm.3850

[4] Ilson DH. Angiogenesis in gastric cancer: hitting the target? Lancet. 2014;383(9911):4–6. doi: 10.1016/S0140-6736(13)61892-9 24094766 10.1016/S0140-6736(13)61892-9

[5] Wilhelm SM, Dumas J, Adnane L, Lynch M, Carter CA, Schütz G, et al Regorafenib (BAY 73–4506): a new oral multikinase inhibitor of angiogenic, stromal and oncogenic receptor tyrosine kinases with potent preclinical antitumor activity. Int J Cancer 2011;129:245–255. doi: 10.1002/ijc.25864 21170960 10.1002/ijc.25864

[6] Davis SL, Eckhardt SG, Messersmith WA, Jimeno A. The development of regorafenib and its current and potential future role in cancer therapy. Drugs Today (Barc). 2013;49(2):105–115. 23462625 10.1358/dot.2013.49.2.1930525

[7] Shirley M, Keating GM. Regorafenib: A Review of Its Use in Patients with Advanced Gastrointestinal Stromal Tumours. Drugs. 2015;75(9):1009–1017. doi: 10.1007/s40265-015-0406-x 25998375 10.1007/s40265-015-0406-x

[8] Chen D, Wei L, Yu J, Zhang L. Regorafenib inhibits colorectal tumor growth through PUMA-mediated apoptosis. Clin Cancer Res. 2014;20(13):3472–84. doi: 10.1158/1078-0432.CCR-13-2944 24763611 10.1158/1078-0432.CCR-13-2944 PMC4079733

[9] Grothey A, Van Cutsem E, Sobrero A, Siena S, Falcone A, Ychou M, et al Regorafenib monotherapy for previously treated metastatic colorectal cancer (CORRECT): an international, multicentre, randomised, placebo-controlled, phase 3 trial. Lancet. 2013;381(9863):303–312. doi: 10.1016/S0140-6736(12)61900-X 23177514 10.1016/S0140-6736(12)61900-X

[10] Li J, Qin S, Xu R, Yau TC, Ma B, Pan H, et al Regorafenib plus best supportive care versus placebo plus best supportive care in Asian patients with previously treated metastatic colorectal cancer (CONCUR): a randomised, double-blind, placebo-controlled, phase 3 trial. Lancet Oncol. 2015;16(6):619–629. doi: 10.1016/S1470-2045(15)70156-7 25981818 10.1016/S1470-2045(15)70156-7

[11] Ohtsu A, Shah MA, Van Cutsem E, Rha SY, Sawaki A, Park SR, et al Bevacizumab in combination with chemotherapy as first-line therapy in advanced gastric cancer: a randomized, double-blind, placebo-controlled phase III study. J Clin Oncol. 2011;29(30):3968–3976. doi: 10.1200/JCO.2011.36.2236 21844504 10.1200/JCO.2011.36.2236

[12] Brenner B, Sarfaty M, Purim O, Kundel Y, Amit L, Abramovich A, et al A Phase Ib/II Study Evaluating the Combination of Weekly Docetaxel and Cisplatin Together with Capecitabine and Bevacizumab in Patients with Advanced Esophago-Gastric Cancer. PLoS One. 2016;11(7):e0157548 PMCID: PMC4938513. doi: 10.1371/journal.pone.0157548 27390847 10.1371/journal.pone.0157548 PMC4938513

[13] Yi JH, Lee J, Lee J, Park SH, Park JO, Yim DS, et al Randomised phase II trial of docetaxel and sunitinib in patients with metastatic gastric cancer who were previously treated with fluoropyrimidine and platinum. Br J Cancer. 2012;106(9):1469–1474. PMCID: PMC3341944. doi: 10.1038/bjc.2012.100 22460270 10.1038/bjc.2012.100 PMC3341944

[14] Garrido M, Fonseca PJ, Vieitez JM, Frunza M, Lacave AJ. Challenges in first line chemotherapy and targeted therapy in advanced gastric cancer. Expert Rev Anticancer Ther. 2014;14(8):887–900. doi: 10.1586/14737140.2014.915194 24953238 10.1586/14737140.2014.915194

[15] Pavlakis N, Sjoquist KM, Martin AJ, Tsobanis E, Yip S, Kang YK, et al Regorafenib for the Treatment of Advanced Gastric Cancer (INTEGRATE): A Multinational Placebo-Controlled Phase II Trial. J Clin Oncol. 2016;34(23):2728–2735. PMCID: PMC5019744. doi: 10.1200/JCO.2015.65.1901 27325864 10.1200/JCO.2015.65.1901 PMC5019744

[16] Burki TK. Progression-free survival with regorafenib in gastric cancer. Lancet Oncol. 2016;17(8):e323. 10.1016/S1470-2045(16)30284-4 27375105

[17] Qiao J, Liu Z, Yang C, Gu L, Deng D. SRF promotes gastric cancer metastasis through stromal fibroblasts in an SDF1-CXCR4-dependent manner. Oncotarget. 2016,7(29):46088–46099. doi: 10.18632/oncotarget.10024 27323859 10.18632/oncotarget.10024 PMC5216783

[18] Izumi D, Ishimoto T, Miyake K, Sugihara H, Eto K, Sawayama H, et al CXCL12/CXCR4 activation by cancer-associated fibroblasts promotes integrin β1 clustering and invasiveness in gastric cancer. Int J Cancer. 2016;138(5):1207–19. doi: 10.1002/ijc.29864 26414794 10.1002/ijc.29864

[19] Lombardi L, Tavano F, Morelli F, Latiano TP, Di Sebastiano P, Maiello E. Chemokine receptor CXCR4: role in gastrointestinal cancer. Crit Rev Oncol Hematol. 2013;88(3):696–705. doi: 10.1016/j.critrevonc.2013.08.005 24120239 10.1016/j.critrevonc.2013.08.005

[20] Domanska UM, Kruizinga RC, Nagengast WB, Timmer-Bosscha H, Huls G, de Vries EG, et al A review on CXCR4/CXCL12 axis in oncology: no place to hide. Eur J Cancer. 2013;49(1):219–30. doi: 10.1016/j.ejca.2012.05.005 22683307 10.1016/j.ejca.2012.05.005

[21] Teicher BA, Fricker SP. CXCL12 (SDF-1)/CXCR4 pathway in cancer. Clin Cancer Res. 2010 6 1;16(11):2927–31. doi: 10.1158/1078-0432.CCR-09-2329 20484021 10.1158/1078-0432.CCR-09-2329

[22] Fuchs CS, Tomasek J, Yong CJ, Dumitru F, Passalacqua R, Goswami C, et al Ramucirumab monotherapy for previously treated advanced gastric or gastro-oesophageal junction adenocarcinoma (REGARD): an international, randomised, multicentre, placebo-controlled, phase 3 trial. Lancet. 2014;383(9911):31–39. doi: 10.1016/S0140-6736(13)61719-5 24094768 10.1016/S0140-6736(13)61719-5

[23] Wilke H, Muro K, Van Cutsem E, Oh SC, Bodoky G, Shimada Y, et al Ramucirumab plus paclitaxel versus placebo plus paclitaxel in patients with previously treated advanced gastric or gastro-oesophageal junction adenocarcinoma (RAINBOW): a double-blind, randomised phase 3 trial. Lancet Oncol. 2014;15(11):1224–1235. doi: 10.1016/S1470-2045(14)70420-6 25240821 10.1016/S1470-2045(14)70420-6

[24] Li J, Qin S, Xu J, Xiong J, Wu C, Bai Y, et al Randomized, Double-Blind, Placebo-Controlled Phase III Trial of Apatinib in Patients With Chemotherapy-Refractory Advanced or Metastatic Adenocarcinoma of the Stomach or Gastroesophageal Junction. J Clin Oncol. 2016;34(13):1448–1454. doi: 10.1200/JCO.2015.63.5995 26884585 10.1200/JCO.2015.63.5995

[25] Riquelme I, Saavedra K, Espinoza JA, Weber H, García P, Nervi B, et al Molecular classification of gastric cancer: Towards a pathway-driven targeted therapy. Oncotarget. 2015;6(28):24750–79. PMCID: PMC4694793. doi: 10.18632/oncotarget.4990 26267324 10.18632/oncotarget.4990 PMC4694793

[26] Yu J, Zhang Y, Leung LH, Liu L, Yang F, Yao X. Efficacy and safety of angiogenesis inhibitors in advanced gastric cancer: a systematic review and meta-analysis. J Hematol Oncol. 2016;9(1):111 PMCID: PMC5070169. doi: 10.1186/s13045-016-0340-8 27756337 10.1186/s13045-016-0340-8 PMC5070169

[27] Janjigian YY, Ku GY, Chou JF, Capanu M, Siebel M, Chalasani SB, et al Phase II study of FOLFOX plus regorafenib (REGO) in patients with unresectable or metastatic esophagogastric (EG) cancer. J Clin Oncol. 2015;33 Suppl:Abstr 4053

[28] Chen LT, Oh DY, Ryu MH, Yeh KH, Yeo W, Carlesi R, et al Anti-angiogenic Therapy in Patients with Advanced Gastric and Gastroesophageal Junction Cancer: A Systematic Review. Cancer Res Treat. 2017 1 3. [Epub ahead of print] 10.4143/crt.2016.176 PMC5654167 28052652

[29] Lee HJ, Song IC, Yun HJ, Jo DY, Kim S. CXC chemokines and chemokine receptors in gastric cancer: from basic findings towards therapeutic targeting. World J Gastroenterol. 2014;20(7):1681–93. doi: 10.3748/wjg.v20.i7.1681 24587647 10.3748/wjg.v20.i7.1681 PMC3930968

[30] Jiang YX, Yang SW, Li PA, Luo X, Li ZY, Hao YX, et al The promotion of the transformation of quiescent gastric cancer stem cells by IL-17 and the underlying mechanisms. Oncogene. 2016. [Epub ahead of print] 10.1038/onc.2016.291 PMC5340802 27524415

[31] Guo ZJ, Yang L, Qian F, Wang YX, Yu X, Ji CD, et al Transcription factor RUNX2 up-regulates chemokine receptor CXCR4 to promote invasive and metastatic potentials of human gastric cancer. Oncotarget. 2016;7(15):20999–21012. doi: 10.18632/oncotarget.8236 27007162 10.18632/oncotarget.8236 PMC4991507

[32] Vandercappellen J, Van Damme J, Struyf S. The role of CXC chemokines and their receptors in cancer. Cancer Lett. 2008; 267(2): 226–244. doi: 10.1016/j.canlet.2008.04.050 18579287 10.1016/j.canlet.2008.04.050

[33] Lee HJ, Kim SW, Kim HY, Li S, Yun HJ, Song KS, et al Chemokine receptor CXCR4 expression, function, and clinical implications in gastric cancer. Int J Oncol 2009; 34(2):473–480. 19148483

[34] Iwasa S, Yanagawa T, Fan J, Katoh R. Expression of CXCR4 and its ligand SDF-1 in intestinal-type gastric cancer is associated with lymph node and liver metastasis. Anticancer Res. 2009; 29(11): 4751–4758. 20032431

[35] Bao W, Fu HJ, Xie QS, Wang L, Zhang R, Guo ZY, et al HER2 interacts with CD44 to up-regulate CXCR4 via epigenetic silencing of microRNA-139 in gastric cancer cells. Gastroenterology.2011; 141(6): 2076–2087.e6. doi: 10.1053/j.gastro.2011.08.050 21925125 10.1053/j.gastro.2011.08.050

[36] He H, Wang C, Shen Z, Fang Y, Wang X, Chen W, et al Upregulated expression of C-X-C chemokine receptor 4 is an independent prognostic predictor for patients with gastric cancer. PLOS One.2013; 8(8): e71864 doi: 10.1371/journal.pone.0071864 23936528 10.1371/journal.pone.0071864 PMC3735563

[37] Oberlin E, Amara A, Bachelerie F, Bessia C, Virelizier JL, Arenzana-Seisdedos F, et al The CXC chemokine SDF-1 is the ligand for LESTR/fusin and prevents infection by T-cell-line-adapted HIV-1. Nature. 1996; 382(6594):833–835. doi: 10.1038/382833a0 8752281 10.1038/382833a0

[38] Fontanella R, Pelagalli A, Nardelli A, D'Alterio C, Ieranò C, Cerchia L, et al A novel antagonist of CXCR4 prevents bone marrow-derived mesenchymal stem cell-mediated osteosarcoma and hepatocellular carcinoma cell migration and invasion. Cancer Lett. 2016;370(1):100–107. doi: 10.1016/j.canlet.2015.10.018 26517945 10.1016/j.canlet.2015.10.018

[39] Jiang C, Fang X, Zhang H, Wang X, Li M, Jiang W, et al AMD3100 combined with triptolide inhibit proliferation, invasion and metastasis and induce apoptosis of human U2OS osteosarcoma cells. Biomed Pharmacother. 2017 2;86:677–685. doi: 10.1016/j.biopha.2016.12.055 28038429 10.1016/j.biopha.2016.12.055

[40] Calinescu AA, Yadav VN, Carballo E, Kadiyala P, Tran D, Zamler D, et al Survival and proliferation of neural progenitor derived glioblastomas under hypoxic stress is controlled by a CXCL12/CXCR4 autocrine positive feedback mechanism. Clin Cancer Res. 2016 8 19. pii: clincanres.2888.2015. [Epub ahead of print] 10.1158/1078-0432.CCR-15-2888 PMC5316506 27542769

[41] Nakamura T, Shinriki S, Jono H, Guo J, Ueda M, Hayashi M, et al Intrinsic TGF-β2-triggered SDF-1-CXCR4 signaling axis is crucial for drug resistance and a slow-cycling state in bone marrow-disseminated tumor cells. Oncotarget. 2015;6(2):1008–19. doi: 10.18632/oncotarget.2826 25504440 10.18632/oncotarget.2826 PMC4359213

[42] Gao DY, Lin TsT, Sung YC, Liu YC, Chiang WH, Chang CC, et al CXCR4-targeted lipid-coated PLGA nanoparticles deliver sorafenib and overcome acquired drug resistance in liver cancer. Biomaterials.2015;67:194–203. doi: 10.1016/j.biomaterials.2015.07.035 26218745 10.1016/j.biomaterials.2015.07.035

[43] Fan D, Ren B, Yang X, Liu J, Zhang Z. Upregulation of miR-501-5p activates the wnt/β-catenin signaling pathway and enhances stem cell-like phenotype in gastric cancer. J Exp Clin Cancer Res. 2016;35(1):177 PMCID: PMC5111270 doi: 10.1186/s13046-016-0432-x 27846906 10.1186/s13046-016-0432-x PMC5111270

[44] Peng Y, Zhang X, Ma Q, Yan R, Qin Y, Zhao Y, et al MiRNA-194 activates the Wnt/β-catenin signaling pathway in gastric cancer by targeting the negative Wnt regulator, SUFU. Cancer Lett. 2017;385:117–127. pii: S0304-3835(16)30666-8. doi: 10.1016/j.canlet.2016.10.035 27810403 10.1016/j.canlet.2016.10.035

[45] Santos JC Carrasco-Garcia E, Garcia-Puga M, Aldaz P, Montes M, Fernandez-Reyes M, de Oliveira CC, et al SOX9 Elevation Acts with Canonical WNT Signaling to Drive Gastric Cancer Progression. Cancer Res. 2016 8 28. [Epub ahead of print] 10.1158/0008-5472.CAN-16-1120 27569216

[46] Zhao S, Wang J, Qin C. Blockade of CXCL12/CXCR4 signaling inhibits intrahepatic cholangiocarcinoma progression and metastasis via inactivation of canonical Wnt pathway. J Exp Clin Cancer Res. 2014;33:103 doi: 10.1186/s13046-014-0103-8 25471741 10.1186/s13046-014-0103-8 PMC4265318

