# Supplementary material for: Regorafenib inhibited gastric cancer cells growth and invasion via CXCR4 activated Wnt pathway
Source: PLoS One. 2017 May 10;12(5):e0177335. doi: 10.1371/journal.pone.0177335 (PMC5425213; doi:10.1371/journal.pone.0177335)
Supplement: S1 Table — (DOC) [file pone.0177335.s003.doc]

**Cytotoxicity assessment of regorafenib on the growth of SGC7901 cell** （$\bar{X}$±S）%

| concentration | Day 1 | Day 2 | Day 3 | Day 4 | Day 5 |
| --- | --- | --- | --- | --- | --- |
| Control | 58.02±4.62 | 90.22±4.11 | 113.88±6.57 | 130.26±6.21 | 144.24±5.71 |
| Reg 5μM | 58.02±4.69  / *p*＞0.05 | 86.22±5.72  / *p*＞0.05 | 104.32±5.98  / *p*＞0.05 | 123.82±4.90  / *p*＞0.05 | 135.86±5.05  /*p*=0.041 |
| Reg 10μM | 58.34±4.46  / *p*＞0.05 | 82.72±5.85  / *p*=0.047 | 98.06±5.66  / *p*=0.041 | 112.68±5.47  / *p*=0.027 | 124.34±5.26  / *p*=0.019 |
| Reg 20μM | 58.16±4.76  / *p*＞0.05 | 75.04±4.85  / *p*=0.032 | 70.5±3.22  / *p*=0.017 | 66.96±3.43  / *p*=0.007 | 62.76±5.12  / *p*=0.005 |
| Reg 40μM | 57.38±4.71  / *p*＞0.05 | 51.2±3.95  / *p*=0.009 | 44.14±3.86  / *p*=0.006 | 36.68±5.56  / *p*＜0.001 | 29.64±6.68  / *p*＜0.001 |

$\bar{X}$, mean; S, SD (Standard Deviation)

**Cytotoxicity assessment of regorafenib on the growth of MKN28 cell** （$\bar{X}$±S）%

| concentration | Day 1 | Day 2 | Day 3 | Day 4 | Day 5 |
| --- | --- | --- | --- | --- | --- |
| Control | 57.08±4.67 | 87.08±5.07 | 107.14±5.06 | 121.14±6.33 | 131.42±5.14 |
| Reg 5μM | 57.98±4.98  / *p*＞0.05 | 86.04±5.38  / *p*＞0.05 | 102.16±5.57  */ p*=0.021 | 118.20±5.35  / *p*＞0.05 | 127.34±5.02  / *p*＞0.05 |
| Reg 10μM | 57.20±5.76  / *p*＞0.05 | 79.66±5.80  / *p*＞0.05 | 95.18±4.65  / *p*=0.036 | 111.50±4.60  / *p*＞0.05 | 118.38±6.97  / *p*=0.047 |
| Reg 20μM | 55.56±5.14  / *p*＞0.05 | 61.62±4.70  / *p*=0.021 | 55.30±4.16  / *p*=0.009 | 55.48±2.04  */ p*=0.006 | 52.68±3.42  */ p*=0.004 |
| Reg 40μM | 58.48±6.68  / *p*＞0.05 | 58.16±5.26  / *p*=0.02 | 52.82±4.84  */ p*=0.008 | 47.96±4.29  */ p*=0.004 | 42.36±5.06  */ p*=0.002 |

$\bar{X}$, mean; S, SD (Standard Deviation).

**Cytotoxicity assessment of regorafenib on the growth of MKN45 cell** （$\bar{X}$±S）%

| concentration | Day 1 | Day 2 | Day 3 | Day 4 | Day 5 |
| --- | --- | --- | --- | --- | --- |
| Control | 55.94±5.81 | 89.94±7.02 | 116.84±5.60 | 134.70±5.23 | 151.60±7.22 |
| Reg 5μM | 56.18±4.60  / *p*＞0.05 | 86.54±5.31  / *p*＞0.05 | 109.70±3.65  / *p*＞0.05 | 127.44±3.86  / *p*＞0.05 | 141.14±7.50  / *p*＞0.05 |
| Reg 10μM | 57.04±5.80  / *p*＞0.05 | 84.62±4.60  / *p*＞0.05 | 106.00±6.25  / *p*＞0.05 | 121.86±6.07  / *p*＞0.05 | 133.56±6.61  / *p*=0.041 |
| Reg 20μM | 55.76±4.80  / *p*＞0.05 | 80.10±6.51  / *p*＞0.05 | 73.44±5.97  */ p*=0.019 | 65.90±6.59  */ p*=0.008 | 60.46±6.15  */ p*=0.003 |
| Reg 40μM | 56.20±5.72  / *p*＞0.05 | 45.58±5.44  / *p*=0.009 | 36.56±5.14  / *p*＜0.001 | 28.50±4.88  */ p*＜0.001 | 18.14±4.38  */ p*＜0.001 |

$\bar{X}$, mean; S, SD (Standard Deviation).
