# Supplementary material for: Regorafenib inhibited gastric cancer cells growth and invasion via CXCR4 activated Wnt pathway
Source: PLoS One. 2017 May 10;12(5):e0177335. doi: 10.1371/journal.pone.0177335 (PMC5425213; doi:10.1371/journal.pone.0177335)
Supplement: S2 Table — (DOC) [file pone.0177335.s004.doc]

**Cell colonies in regorafenib groups (20µmol/L) in soft agar assay（$\bar{X}$±S）**

| Cells | Control | Reg 20μM | *P* |
| --- | --- | --- | --- |
| SGC7901 | 176±26.63 | 32±9.54 | *p*<0.001 |
| MKN28 | 125±27.06 | 8.33±3.51 | *p*<0.001 |
| MKN45 | 201±31.05 | 43.33±14.50 | *p*=0.003 |

$\bar{X}$, mean; S, SD (Standard Deviation).
