# Supplementary material for: Regorafenib inhibited gastric cancer cells growth and invasion via CXCR4 activated Wnt pathway
Source: PLoS One. 2017 May 10;12(5):e0177335. doi: 10.1371/journal.pone.0177335 (PMC5425213; doi:10.1371/journal.pone.0177335)
Supplement: S3 Table — (DOC) [file pone.0177335.s005.doc]

**Number of invasive cells with regorafenib at the concentration of 10μmol/L or 20μmol/L** （±S）

| Cells | Control | Reg 10μM | Reg 20μM |
| --- | --- | --- | --- |
| SGC7901 | 17±3.00 | 10±2.65  / *p*=0.021 | 3±1.00  / *p*=0.001 |
| MKN28 | 17±3.61 | 10.33±2.08  / *p*=0.022 | 2.67±0.58  / *p*=0.001 |
| MKN45 | 25±4.00 | 9±2.65  / *p*=0.006 | 3.33±1.15  / *p*=0.001 |

$\bar{X}$, mean; S, SD (Standard Deviation).
