# Supplementary material for: Regorafenib inhibited gastric cancer cells growth and invasion via CXCR4 activated Wnt pathway
Source: PLoS One. 2017 May 10;12(5):e0177335. doi: 10.1371/journal.pone.0177335 (PMC5425213; doi:10.1371/journal.pone.0177335)
Supplement: S4 Table — (DOC) [file pone.0177335.s006.doc]

**The mRNA levels of CXCR4 of SGC7901 Cells treated with regorafenib at different concentration for 24 hours or at different times at the concentration of 20µmol/L** （$\bar{X}$±S）%

| concentration | Control | Reg 5μM | Reg 10μM | Reg 20μM | Reg 40μM |
| --- | --- | --- | --- | --- | --- |
| CXCR4 level | 99.67±4.51 | 97.33±6.03 | 77.67±8.08 | 27.00±6.25 | 15.67±6.66 |
| time | 0 hours | 8 hours | 16 hours | 24 hours | 36 hours |
| CXCR4 level | 101±3.61 | 99.67±6.03 | 73.67±7.02 | 26.00±6.56 | 24.33±4.73 |

$\bar{X}$, mean; S, SD (Standard Deviation).
