# Supplementary material for: Regorafenib inhibited gastric cancer cells growth and invasion via CXCR4 activated Wnt pathway
Source: PLoS One. 2017 May 10;12(5):e0177335. doi: 10.1371/journal.pone.0177335 (PMC5425213; doi:10.1371/journal.pone.0177335)
Supplement: S5 Table — (DOC) [file pone.0177335.s007.doc]

**Number of invasive cells with different test treatments** （±S）

| Groups | Control | CXCL12 | CXCL12  +CXCR4+ | Reg | CXCL12  +Reg | CXCL12+Reg+CXCR4+ |
| --- | --- | --- | --- | --- | --- | --- |
| SGC7901l | 15.67±2.52 | 21.33±4.04  / *p*=0.037 | 27.67±4.16  / *p*=0.013 | 3.00±1.00  / *p*=0.002 | 6.67±1.53  / *p*=0.014 | 7.00±2.00  / *p*=0.015 |
| MKN45 | 20.00±3.61 | 26.00±3.00  / *p*=0.039 | 31.00±3.61  / *p*=0.017 | 3.67±0.58  / *p*=0.002 | 7.33±2.08  / *p*=0.008 | 8.00±2.00  / *p*=0.01 |

$\bar{X}$, mean; S, SD (Standard Deviation).
