# Supplementary material for: Regorafenib inhibited gastric cancer cells growth and invasion via CXCR4 activated Wnt pathway
Source: PLoS One. 2017 May 10;12(5):e0177335. doi: 10.1371/journal.pone.0177335 (PMC5425213; doi:10.1371/journal.pone.0177335)
Supplement: S6 Table — (DOC) [file pone.0177335.s008.doc]

**The activity of TOP and FOP flash in gastric cancer cells treated with regorafenib or CXCR4 overexpression** （$\bar{X}$±S）

| **SGC7901** | Control | Reg 20μM | CXCR4+ |
| --- | --- | --- | --- |
| TOPFLASH | 0.99±0.05 | 0.50±0.06  / *p*=0.011 | 2.16±0.22  / *p*=0.012 |
| FOPFLASH | 0.99±0.05 | 0.99±0.04 | 0.99±0.08 |
|  |  |  |  |
| **MKN28** | Control | Reg 20μM | CXCR4+ |
| TOPFLASH | 0.99±0.06 | 0.74±0.08  / *p*=0.038 | 1.88±0.20  / *p*=0.017 |
| FOPFLASH | 0.98±0.05 | 0.95±0.04 | 0.97±0.06 |
|  |  |  |  |
| **MKN45** | Control | Reg 20μM | CXCR4+ |
| TOPFLASH | 0.98±0.05 | 0.60±0.06  / *p*=0.026 | 2.00±0.24  / *p*=0.013 |
| FOPFLASH | 0.99±0.06 | 0.98±0.05 | 0.97±0.07 |

$\bar{X}$, mean; S, SD (Standard Deviation).
