# Supplementary material for: Regorafenib inhibited gastric cancer cells growth and invasion via CXCR4 activated Wnt pathway
Source: PLoS One. 2017 May 10;12(5):e0177335. doi: 10.1371/journal.pone.0177335 (PMC5425213; doi:10.1371/journal.pone.0177335)
Supplement: S7 Table — (DOC) [file pone.0177335.s009.doc]

**The expression of Wnt target genes in gastric cancer cells with CXCR4 overexpression or treated with regorafenib** （$\bar{X}$±S）

| **CTNNB1** | Control | Reg 20μM | CXCR4+ |
| --- | --- | --- | --- |
| SGC7901 | 0.99±0.06 | 0.47±0.06 */ p*=0.012 | 2.08±0.22 */ p*=0.011 |
| MKN28 | 0.98±0.05 | 0.66±0.06 */ p*=0.023 | 1.67±0.16 */ p*=0.027 |
| MKN45 | 1.02±0.04 | 0.52±0.06 */ p*=0.015 | 1.85±0.14 */ p*=0.017 |
|  |  |  |  |
| **CD44** | Control | Reg 20μM | CXCR4+ |
| SGC7901 | 0.99±0.05 | 0.52±0.06 */ p*=0.013 | 1.90±0.10 */ p*=0.014 |
| MKN28 | 0.97±0.04 | 1.00±0.06 | 1.08±0.10 |
| MKN45 | 0.99±0.04 | 0.61±0.06 */ p*=0.026 | 1.75±0.12 */ p*=0.017 |
|  |  |  |  |
| **CD31** | Control | Reg 20μM | CXCR4+ |
| SGC7901 | 1.00±0.03 | 0.60±0.07 */ p*=0.019 | 1.92±0.15 */ p*=0.014 |
| MKN28 | 0.98±0.05 | 0.74±0.09 */ p*=0.039 | 1.51±0.12 */ p*=0.033 |
| MKN45 | 0.98±0.04 | 0.61±0.07 */ p*=0.029 | 1.78±0.12 */ p*=0.017 |
|  |  |  |  |
| **CCND1** | Control | Reg 20μM | CXCR4+ |
| SGC7901 | 0.99±0.05 | 0.63±0.07 */ p*=0.019 | 1.78±0.13 */ p*=0.018 |
| MKN28 | 1.03±0.05 | 0.95±0.05 | 1.04±0.08 |
| MKN45 | 1.04±0.06 | 0.54±0.07 */ p*=0.013 | 1.89±0.11 */ p*=0.015 |

$\bar{X}$, mean; S, SD (Standard Deviation).
