# Supplementary material for: Regorafenib inhibited gastric cancer cells growth and invasion via CXCR4 activated Wnt pathway
Source: PLoS One. 2017 May 10;12(5):e0177335. doi: 10.1371/journal.pone.0177335 (PMC5425213; doi:10.1371/journal.pone.0177335)
Supplement: S8 Table — (DOC) [file pone.0177335.s010.doc]

**Clinical outcomes of recent trials of regorafenib in advanced gastric and gastroesophageal junction cancer**

| **Author and trial** | **Phase** | **Line** | **Primary endpoint** | **Regimens** | **N** | **Outcomes** |
| --- | --- | --- | --- | --- | --- | --- |
| Pavlakis et al. INTEGRATE [15] | Ⅱ | Ⅱ-Ⅲ | PFS | Regorafenib  Vs Placebo | 97  50 | mOS: 5.8 mo vs 4.5 mo (HR = 0.74, 95% CI (0.51-1.08) P =0.11)  mPFS: 2.6 mo vs 0.9 mo (HR = 0.40, 95% CI (0.28-0.59) P< 0.0001)  CR or PR: 3 patients (95% CI, 1% to 9%) vs 1 patient (95% CI, 0% to 11%)  Clinical benefit at 2 months: 44 patients (95% CI, 36% to 57%) vs 9 patients (95% CI, 9% to 31%) |
| Janjigian et al [27].  NCT01913639 | Ⅱ | ⅠSingle-arm | PFS | Regorafenib  +FOLFOX | 36 | This study is ongoing  mOS: NR; PFS: 17 of 36 patients progression free at 6mo  PR: 56%,95% CI 0.38-0.73 |

N, number (of patients); mOS, median overall survival; HR (95% CI), hazard ratio (95% confidence interval); mPFS, median progression-free survival; FOLFOX, folinic acid, 5-fluorouracil, and oxaliplatin; NR, not reported; RR, response rate; PR, partial response; CR, complete response; SD, stable disease.
